# Diet characterization and a preliminary investigation into trophic niche placement for an endangered lake sturgeon (*Acipenser fulvescens*) population in the Saskatchewan River, SK, Canada

**DOI:** 10.1371/journal.pone.0206313

**Published:** 2018-11-01

**Authors:** David P. Braun, Iain D. Phillips, Lushani Nanayakkara, Björn Wissel

**Affiliations:** 1 Department of Biology, University of Regina, Regina, Saskatchewan, Canada; 2 Water Security Agency, Saskatoon, Saskatchewan, Canada; Tanzania Fisheries Research Institute, UNITED REPUBLIC OF TANZANIA

## Abstract

All Canadian lake sturgeon (*Acipenser fulvescens*) populations are listed under the Committee on the Status of Endangered Wildlife in Canada (COSEWIC) due to their complex life history, loss of habitat and negative interactions with anthropomorphic activities. Lake sturgeon diets vary considerably across their range and depend on the local benthic invertebrate fauna, substrata, and competition with congener species. Hence, determining population-specific prey assemblages is a vital contribution to effective conservation efforts. We used carbon and nitrogen stable isotopes to identify lake sturgeon prey preferences for juvenile (<100 cm) and adult (>100 cm) age classes and their trophic niche in the Saskatchewan River, SK, Canada. In this system, lake sturgeon were the top predator within the benthic food web with no direct competition for preferred prey items from congener species. Their diet was dominated by crayfish (49.1± 6.4%) and snails (36.3 ± 5.5%), with no significant differences between age classes. Furthermore, lake sturgeon favoured deep rocky pools throughout the river; a habitat where crayfish and snails are typically found. Therefore, conservation efforts should be directed at preserving these habitats for the residing benthic invertebrate community, and the lake sturgeon’s ability to access them.

## Introduction

Sturgeon, Acipenseridae, is one of the most prevalent and charismatic families of fishes worldwide, inhabiting estuaries, rivers, near-shore oceanic environments and inland seas of the northern hemisphere [1]. Of the 25 extant species, white sturgeon (*Acipenser transmontanus* [Richardson, 1836]), green sturgeon (*Acipenser medirostris* [Ayres, 1854]), Atlantic sturgeon (*Acipenser oxyrhynchus* [Mitchill, 1815]), shortnose sturgeon (*Acipenser brevirostrum* [Lesueur, 1818 non Heckel, 1836]), and lake sturgeon (*Acipenser fulvescens* [Rafinesque, 1817]) are native to Canada. Among them, lake sturgeon is the only endemic potamodromous species, with a range spanning from Alberta to Quebec [1]. In 2006, the Committee on the Status of Endangered Wildlife in Canada (COSEWIC) listed the Saskatchewan River populations, along with four other populations, as endangered, two of special concern, and one threatened [2].

Many sturgeon species are susceptible to anthropomorphic alterations to their habitats due to their unique life-history traits. For example, lake sturgeon populations are less resilient to over-exploitation due to a protracted juvenile stage; erratic spawning once every five years for females, and every second year for males; plus migrating distances exceeding 200 km between overwintering and spawning locations [3, 4, 5, 6]. Anthropogenic impacts, such as blocked migratory routes by hydroelectric dams, water pollution, and extensive caviar harvesting in the late 1800’s and early 1900’s, have resulted in significant reductions of lake sturgeon populations [5, 7, 8, 9].

To support the remaining populations, long-term and continuous availability of suitable prey/habitat is required. Due to the emerging belief that diet/habitat characterization may not be consistent among populations, increased study efforts are necessary to characterize population-specific prey sources, spawning habitats, and overwintering habitats, with the hope of constructing effective conservation plans for specific watersheds [10]. Lake sturgeon are generally opportunistic benthic feeders that utilize their morphologically-distinct extending mouth situated at the end of a proboscis to consume prey [5]. Previous studies in habitats with coarse (ie. gravel, cobble, boulders) or fine (ie. sand, silt, clay) substrate have identified diverse diet items including mayflies (Ephemeroptera [Hyatt and Arms, 1891]), crane flies (Tipulidae [Latreille,1802]), caddisflies (Trichoptera [Kirby, 1813]), chironomids (Chironomidae [Meigen, 1803]), gammarids (Gammaridae [Leach, 1813]), mussels (Bivalvia [Linnaeus, 1758]), snails (Gastropoda [Cuvier, 1795]), crayfish (Decapoda [Latrielle, 1802]) [11, 12, 13, 14, 15, 16, 17, 18, 19], and occasionally small fishes [14, 17, 18]. Yet, diets of individual lake sturgeon populations are often more limited, depending on regionally available food sources [11, 12, 13, 14, 15, 16, 17, 18, 19].

With destructive techniques of diet reconstruction (i.e., stomach content analyses) no longer acceptable for endangered species, stable isotope analyses has become a powerful non-invasive sampling technique that can accurately determine prey preferences. Stable isotopes have increasingly become more prevalent in freshwater studies, elucidating food-web structures, prey selection, and habitat use [20, 21, 22]. Specifically, carbon (δ^13^C) and nitrogen (δ^15^N) stable isotopes are used because of their ability to describe trophic position and prey choices for predators. Values of δ^13^C vary substantially between primary producers with different photosynthetic pathways (C3 vs. C4 plants), terrestrial versus aquatic primary producers, and in lentic and lotic aquatic environments [21, 23, 24, 25]. Since consumers incorporate the δ^13^C of their prey with minimal fractionation during trophic transfers [26], δ^13^C can identify sources of dietary carbon and their location. On the other hand, δ^15^N is an indicator of trophic position because of its predictable fractionation (2–4%) during trophic transfers [21, 26]. Beyond determining the number of trophic levels, the δ^15^N values of primary consumers can establish stable isotope baselines for a food web or a specific location within a watershed [27, 28]. Comparing these baselines can identify if spatial consistency of stable isotope baselines exists within a watershed, or among locations [27]. However, the actual determination of specific prey items consumed is commonly based on mathematical mixing models that quantify the relative contributions of individual prey and associated uncertainties [29, 30, 31].

In this study, we used δ^13^C and δ^15^N stable isotopes to determine lake sturgeon’s trophic position within the Saskatchewan River, Canada. Specifically; we 1) analyzed temporal and spatial variability of lake sturgeon δ^13^C and δ^15^N; 2) identified and quantified common prey sources for juveniles and adults; and, 3) assessed potential feeding competition between lake sturgeon and congener species with overlapping distributions. Together with previous characterizations of spawning and overwintering habitats [10], and migration behavior [6], this study provides much needed information on the feeding behavior of lake sturgeon to develop effective conservation plans.

## Materials and methods

### Study site

**Fig 1:**
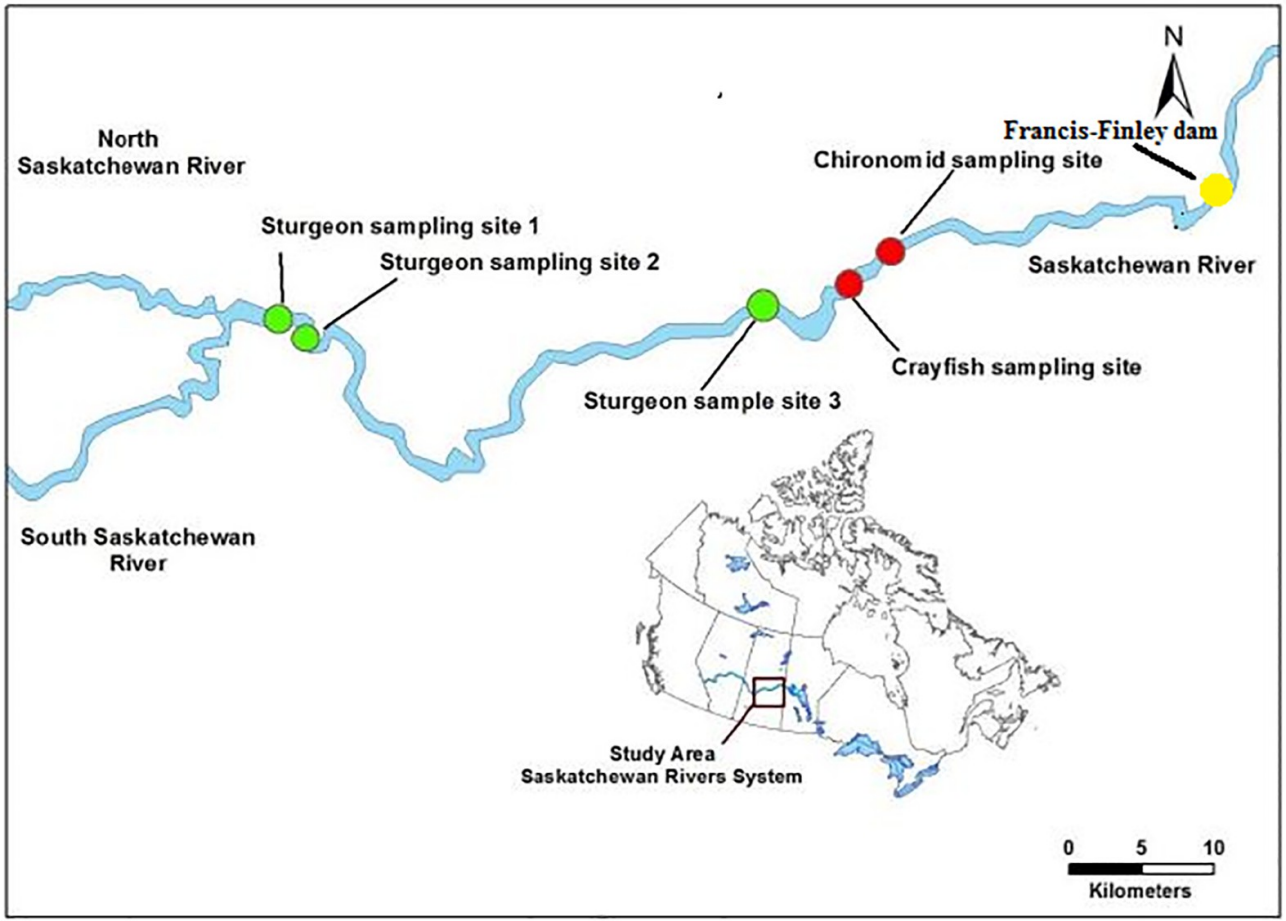
Map of the study area in the Saskatchewan River system, Saskatchewan, Canada. Site 1 is located at the confluence of the North and South Saskatchewan Rivers, forming the Saskatchewan River, site 2 is three kilometres downstream from the confluence on the Saskatchewan River, and site 3 is 60 kilometres downstream from the confluence. Crayfish and Chironomid sampling sites are located in Codette Reservoir.

The study area encompassed a section of the Saskatchewan River, Saskatchewan, Canada spanning from the confluence of the North Saskatchewan and South Saskatchewan Rivers (Latitude: 53.235187; Longitude: -105.08294779031895) to Codette Reservoir, formed by the Francis-Finley dam in Nipawin, Saskatchewan, Canada (Latitude: 53.31808; Longitude: -104.04179185266574) (Fig 1). We focused on three lotic locations (site 1, 2 and 3) to collect lake sturgeon and benthic macroinvertebrates samples. Each site was approximately 280 m wide, 10 m deep, and with a mixed substrate of cobble (65–200 mm) and boulder (> 250 mm). Additionally, fourteen profundal zone sites, and three littoral sites were sampled in Codette Reservoir for benthic macroinvertebrates (Fig 1). The profundal zone sites exceeded depths of 30 m, while the littoral sites were 3–5 m deep. Moreover, both had sand, clay, and silt substrate [10]. The riparian zones for both locations were mainly C3 grasses and herbaceous trees, surrounded by Aspen Parkland and fragmented agriculture. All the sampling locations were located on public land and required a Scientific Collection permit from the Saskatchewan Ministry of Environment.

### Lake sturgeon and companion fish sampling

We collected lake sturgeon samples from May to October 2009, June to September 2010, and May to October 2011 at sites 1, 2, and 3 using two methods: rod and reel, and trotlines. Six rods were used daily over 3-hour intervals at each site. Rods were equipped with a size 4/0 J-hook, 10 g weight, 100 pound test line, and nightcrawler worms (*Lumbricus terrestris* [Linnaeus, 1758]) for bait. When a lake sturgeon was caught, girth, length, and weight were recorded. It was then fitted with a PIT identification tag, and fin clips were taken for stable isotope analysis. In addition, we used three 10 m trotlines containing 10 size 4/0 circle hooks baited with approximately 16 cm^2^ chunks of white sucker flesh (*Catostomus commersonii* [Lacepède, 1803]) at each site. Lines were checked in the morning and evening; lake sturgeon and companion fishes caught were processed as described above. Originally (in 2009 and 2010), bycatch (i.e. companion fishes) were not retained because the study design and sampling techniques focused on lake sturgeon only. However, in 2011 all bycatch captured using lake-sturgeon protocols was retained for further food-web structure analyses. Euthanasia techniques performed by experienced personnel on bycatch in 2011 followed the Canadian Council on Animal Care guidelines via a stunning blow to the head, followed by pithing of the brain [32]. Samples were placed on ice and frozen until processed for further analysis.

Before commencement of our field work, the President’s Committee on Animal Care (PCAC) at the University of Regina reviewed our research and confirmed our sampling procedures were in compliance with institutional and the Canadian Council on Animal Care (CCAC) standards (PCAC Protocol number: AUP 09–10).

### Benthic macroinvertebrate sampling

Benthic macroinvertebrates were collected in August 2010 using four methods: 500 micron D-frame nets, Peterson grabs (30 cm×30 cm), minnow gee traps, and free diving. Standard kick and sweep techniques were applied for collecting macroinvertebrate samples at littoral and riffle sites with D-frame nets [33]. A Peterson Grab was used at Codette Reservoir’s profundal zone sites and at the three lake sturgeon sampling sites. Three sediment grabs were administered at each site and each grab was carefully sieved through a 500 micron mesh, with the remaining benthic macroinvertebrates collected for stable isotope analysis. To capture virile crayfish (*Orconectes virilis* [Hagen 1870]), minnow gee traps were deployed for 80.5 hrs, and baited with stewing beef at littoral sites in Codette Reservoir and the lake sturgeon sampling sites. Lastly, bivalve species at sites 1, 2, 3, and Codette Reservoir’s littoral sites; were collected via free diving with snorkel and mask. Macroinvertebrates were frozen until analysis. Before stable isotope analyses, specimens were rinsed in deionized water, enumerated, and identified to the lowest possible taxonomic level.

### Stable isotope analysis

Stable isotope analyses were conducted for lake sturgeon fin clips [34], lateral muscle tissue of goldeye (*Hiodon alsoides* [Rafinesque, 1819]), longnose sucker (*Catostomus catostomus* [J.R. Forster, 1773]), shorthead redhorse (*Moxostoma macrolepidotum* [Lesueur, 1817]), walleye (*Sander vitreus* [Mitchill, 1818]), and white sucker [20]. Shell and carapace-free muscle tissue was analyzed for caddisflies, chironomids, mayflies, virile crayfish (*Orconectes virilise* [Hagen, 1870]), fatmucket (*Lampsilis siliquoidea* [Rafinesque, 1820]), giant floater (*Pyganodon grandis* [Say, 1829]), white heelsplitter (*Lasmigona complanata* [Barnes, 1823]), *Lymnaea* (*Lymnaea sp*. [Lamarck, 1799]), *Physa* (*Physa sp*. [Draparnaud, 1801]) and *Planorbella* (*Planorbella sp*. [Haideman, 1843]). Samples were thawed, dried at 50°C, homogenized, weighed to 0.5–1.0 mg, and enclosed in tin capsules for analysis. Samples were combusted in a Costech 4010 Elemental Analyzer connected to a Thermoquest Delta V Isotope Ratio Mass Spectrometer via a ConFlo IV interface at the Institute of Environmental Change and Society (IECS) at the University of Regina. Internal laboratory standards were bovine liver and wheat flour. Carbon and nitrogen stable isotope values were expressed in the delta notation (δ) and compared to atmospheric N_2_ and Vienne Pee Dee Belemnite for nitrogen and carbon stable isotopes, respectively. Random samples were run in duplicate for replicability of results for a specific sample matrix. All duplicates (n = 97) were within 0.2% for both δ^13^C and δ^15^N. C:N-based lipid corrections for δ^13^C were conducted according to equations outlined in [34].

### Diet composition analysis

We conducted diet analysis for lake sturgeon using the MixSIAR package in R [35]. MixSIAR is a model platform that incorporates Bayesian mixing model theory, facilitating more robust biotracer mixing model analysis such as diet composition analysis using stable isotopes [34]. Prior to applying the mixing model framework, sturgeon were classified into two age classes; juveniles (<100 cm) and adults (>100 cm) [36]. For this study, we only included data for individuals collected in late summer months (July and August) of 2010 to match the timing of macroinvertebrate prey item collections in August 2010. Among prey groups, the three snail taxa (*Lymnea*, *Physa*, and *Planorbella*) were combined into one ‘snail’ category because there were no significant isotopic differences among them and all taxa have similar ecological niches [37]. Sturgeon diet composition was analyzed using a two-isotope mixing model (δ^13^C and δ^15^N), with eight potential prey items. Prey items were selected based on their abundance in the study area and previous studies that identifies them as potential lake sturgeon diet [11, 12, 13, 14, 15, 16, 17, 18, 19]. The final eight diet sources were caddisfly, chironimid, crayfish, fatmucket, giant floater, mayfly, white heelsplitter, and snails. To the best of our knowledge there are no existing diet-tissue discrimination factors specific to lake sturgeon. Hence, we used the following trophic fractionation factors for all diet sources: δ^13^C 0 ± 0.5‰ and δ^15^N 3.5 ± 0.5‰. We did not include site-specific information as preliminary analyses showed no site-specific differences in stable isotope values for juvenile or adult lake sturgeon. The model was initialized using an uninformed prior where all diet sources are equally likely, and source isotope values were entered into the model as means + SD. The analysis utilized 3 Markov Chain Monte Carlo chains with the following specifications, chain length = 300,000, burn-in = 200,000, thinning = 100. We ran two model configurations; 1) Diet estimation by age class and 2) Diet estimation for the entire population. We included both process error and residual error in the model parameters to obtain the most robust results possible [35, 38]. Model convergence was checked using both Gelman-Rubin and Gewke diagnostic plots [35].

### Statistical analysis

Two-way ANOVA’s were used to test for statistical differences between δ^13^C and δ^15^N isotopic values of lake sturgeon caught in different years and months. Additional one-way ANOVA’s were used to determine significant difference among capture locations for lake sturgeon within years and macroinvertebrate species caught across sampling locations in 2010. Isotopic differences between juvenile (0–100 cm) and adult (>100 cm) age groups were tested using a t-test. Finally, δ^13^C and δ^15^N values for dietary groups and prey species within each group were compared using one-way ANOVA’s. All analyses met the requirements of normality and homoscedasticity without data transformation. Analyses were conducted in IBM SPSS Statistics version 21.

## Results

### Lake sturgeon temporal, ontogenetic, and spatial differences

In 2009, 2010, and 2011 we captured 111, 201 and 138 lake sturgeon, respectively. The highest catch rates were recorded in July and August (n = 130 and 139, respectively) and the lowest in June (n = 45). Mean δ^13^C values in 2009, 2010, and 2011 were -24.7 ± 1.1‰, -24.9 ±1.1‰, and -26.2 ± 1.5‰, respectively, with 2011 values significantly depleted in δ^13^C relative to the previous years (p < 0.05). In all years we observed a gradual depletion in δ^13^C from June to September of about 1‰, resulting in significant differences between June/July (p < 0.05) and August/September (p < 0.05). There was no significant year by month interaction (p > 0.05). Mean δ^15^N values in 2009, 2010, and 2011 were 14.4 ± 0.9‰, 15.6 ± 0.7‰, and 15.1 ± 0.9‰, respectively, with 2009 values significantly depleted in δ^15^N relative to the following years (p < 0.05). There were no significant month or year by month interaction effects (p > 0.05). No significant differences (p > 0.05) were observed for lake sturgeon δ^13^C or δ^15^N among sampling locations within any years.

To assess any ontogenetic dietary changes, carbon and nitrogen stable isotopes were measured for 57 juvenile and 81 adult lake sturgeon (July and August 2010). Juveniles had a mean δ^13^C value of -26.1 ± 0.15‰ and a mean δ^15^N value of 15.8 ± 0.10‰, and adults had values of -25.9 ± 0.12‰ and 15.5 ± 0.08‰, respectively. Del^13^C values were not significantly different between adults and juveniles (p > 0.05), while, despite the relatively small overall difference (0.36‰), δ^15^N values were significantly different (p < 0.05).

### Macroinvertebrate community

**Fig 2:**
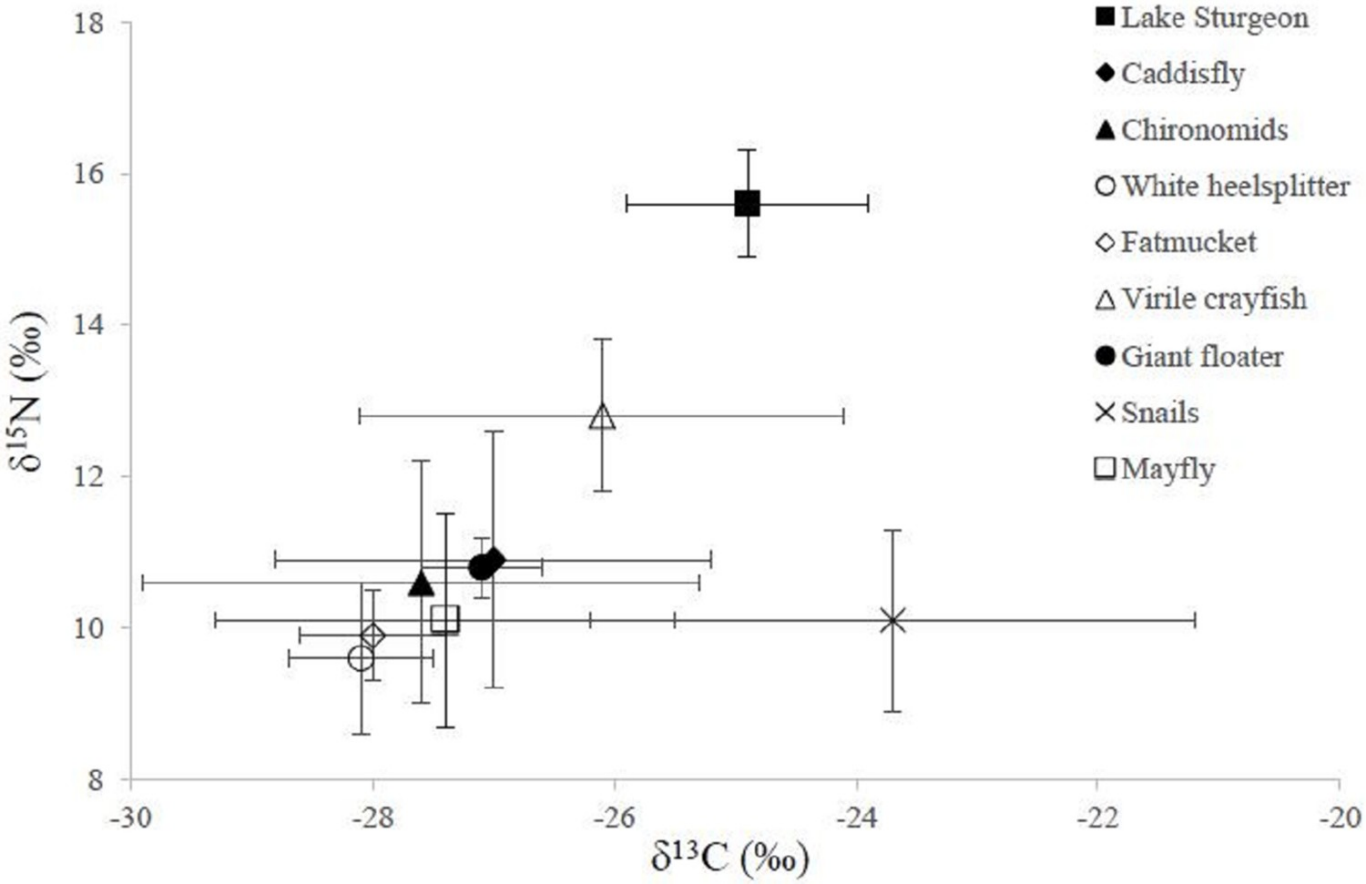
Mean δ^13^C (x-axis) and mean δ^15^N (y-axis) stable isotope values with standard deviations for lake sturgeon and prey items included in the mixing model. All lake sturgeon and prey item included in analysis were caught between July and August 2010.

Twenty four taxa were identified during our study (August 2010), however only ten had abundances (n > 5) sufficient to be considered as potential prey items for lake sturgeon (Table 1). Although ten prey items were identified, the three snail species were entered into the model as one prey item because there were no significant differences between their δ^13^C (p > 0.05) or δ^15^N (p > 0.05) values, and they occupied the same feeding guild. Therefore, only eight prey items were used in the mixing models (Table 2). Of the benthic macroinvertebrates analyzed, crayfish had the highest relative abundance with 64 individuals, highest mean δ^15^N value (12.8 ± 1.01‰), and the second highest mean δ^13^C (-26.1 ± 1.99‰). Snails had the highest mean δ^13^C (-23.7 ± 2.50‰), but their δ^15^N value was intermediate among the prey groups (10.1 ± 1.21‰). The remainder of the prey groups analyzed had mean δ^13^C values below -27‰, with the lowest value being expressed in white heelsplitters (-28.1 ± 0.57‰). White heelsplitters also had the smallest number of individuals collected (n = 7) for the prey included in the mixing model (Table 1). Spatial comparison of baseline stable isotope ratios was done by assessing δ^13^C and δ^15^N of fatmuckets (i.e. mussel), the only filter feeding species collected at each lake sturgeon sampling location. We found no significant differences for mean δ^13^C (p > 0.05) and δ^15^N (p > 0.05) for fatmuckets between locations. Additionally, we found no significant differences between mean δ^13^C (p > 0.05) or δ^15^N (p > 0.05) between sampling sites for all other potential prey items. During this time period (July and August 2010) we caught a total of 138 lake sturgeon with mean δ^13^C values of -24.9 ± 1.0‰ and mean δ^15^N values of 15.6 ± 0.7‰, which were used for the mixing model analyses (Fig 2).

**Table 1:** Sample size and mean δ^13^C and δ^15^N for all benthic macroinvertebrates included in the lake sturgeon diet analysis. Benthic macroinvertebrates were identified to the lowest possible level.

Benthic macroinvertebrate	Sample size	Mean corrected δ^13^C value with standard deviation (‰)	Mean δ^15^N value with standard deviation (‰)
Caddisfly	13	-27.0 ± 1.84	10.9 ± 1.65
Chironomid	18	-27.6 ± 2.29	10.6 ± 1.59
Fatmucket	27	-28.0 ± 0.63	9.9 ± 0.60
Giant floater	23	-27.1 ± 0.46	10.8 ± 0.39
Mayfly	18	-27.3 ± 1.87	10.6 ± 2.32
Snails^§^	16	-23.7 ± 2.50	10.1 ± 1.21
Virile crayfish	64	-26.1 ± 1.99	12.8 ± 1.01
White heelsplitter	7	-28.1 ± 0.57	9.6 ± 0.97

^§^Snails group is made up of *Lymnea* sp., *Physa* sp., and *Planorbella* sp.

**Table 2:** Sample collection method and sampling locations for all macroinvertebrates that were included in the lake sturgeon diet analysis. Macroinvertebrates were identified to the lowest possible level.

Benthic macroinvertebrate	Sample collection method^a^	Macroinvertebrate sampling locations^b^
Caddisfly	D-frame nets	Sturgeon sampling sites 1,2, and 3
Chironomid	Peterson grabs	Codette Reservoir—Profundal sampling sites Sturgeon sampling sites 1,2, and 3
Fatmucket	Free diving	Codette Reservoir—Littoral sampling sites Sturgeon sampling sites 1, 2, and 3
Giant floater	Free diving	Codette Reservoir -Littoral sampling sites Sturgeon sampling site 2
Mayfly	D-frame nets Peterson grabs	Codette Reservoir—Littoral sampling sites Sturgeon sampling sites 1,2, and 3
Snails^c^	D-frame nets Peterson grabs	Codette Reservoir -Littoral and Profundal sampling sites Sturgeon sampling site 2
Virile crayfish	Minnow Gee-traps	Codette Reservoir—Littoral sampling sites Sturgeon sampling site 2
White heelsplitter	Free diving	Codette Reservoir—Littoral sampling sites Sturgeon sampling sites 1 and 2

^a^All sampling techniques we executed at all sampling locations with the exception of the Codette Reservoir profundal sampling sites. These locations where solely sampled using a Peterson grab.

^b^No significant differences between sampling sites were reported for δ^13^C (p > 0.05) or δ^15^N (p > 0.05) values for macroinvertebrate taxa included in the lake sturgeon diet analysis.

^c^Snails group is made up of *Lymnea* sp., *Physa* sp., and *Planorbella* sp.

### Mixing models

**Fig 3:**
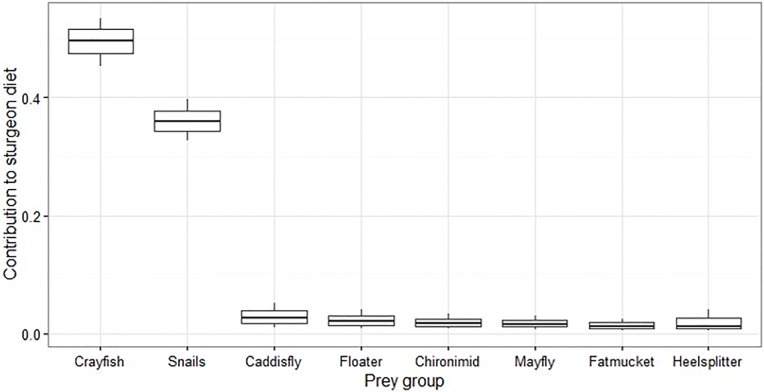
The relative contribution of each prey source to lake sturgeon diets in the Saskatchewan River that were included in the mixing model. Proportional diet contribution is on the y-axis and prey groups are on the x-axis.

Based on our mixing model analyses for summer 2010, we found no significant difference between diet compositions of juvenile and adult lake sturgeon (p<0.05). Therefore, we limit our analyses based on the results from the overall population model. Accordingly, lake sturgeon diets were predominantly composed of crayfish (49.1± 6.4%) and snails (36.3 ± 5.5%), followed by caddisfly (3.7 ± 3.8%), giant floater (2.8± 2.5%), chironimid (2.4 ± 2.2%), mayfly (2.2 ± 2.0%), fatmucket (1.8 ± 1.6%), and white heelsplitter (1.6 ± 1.5%) (Fig 3).

### Companion fish

**Fig 4:**
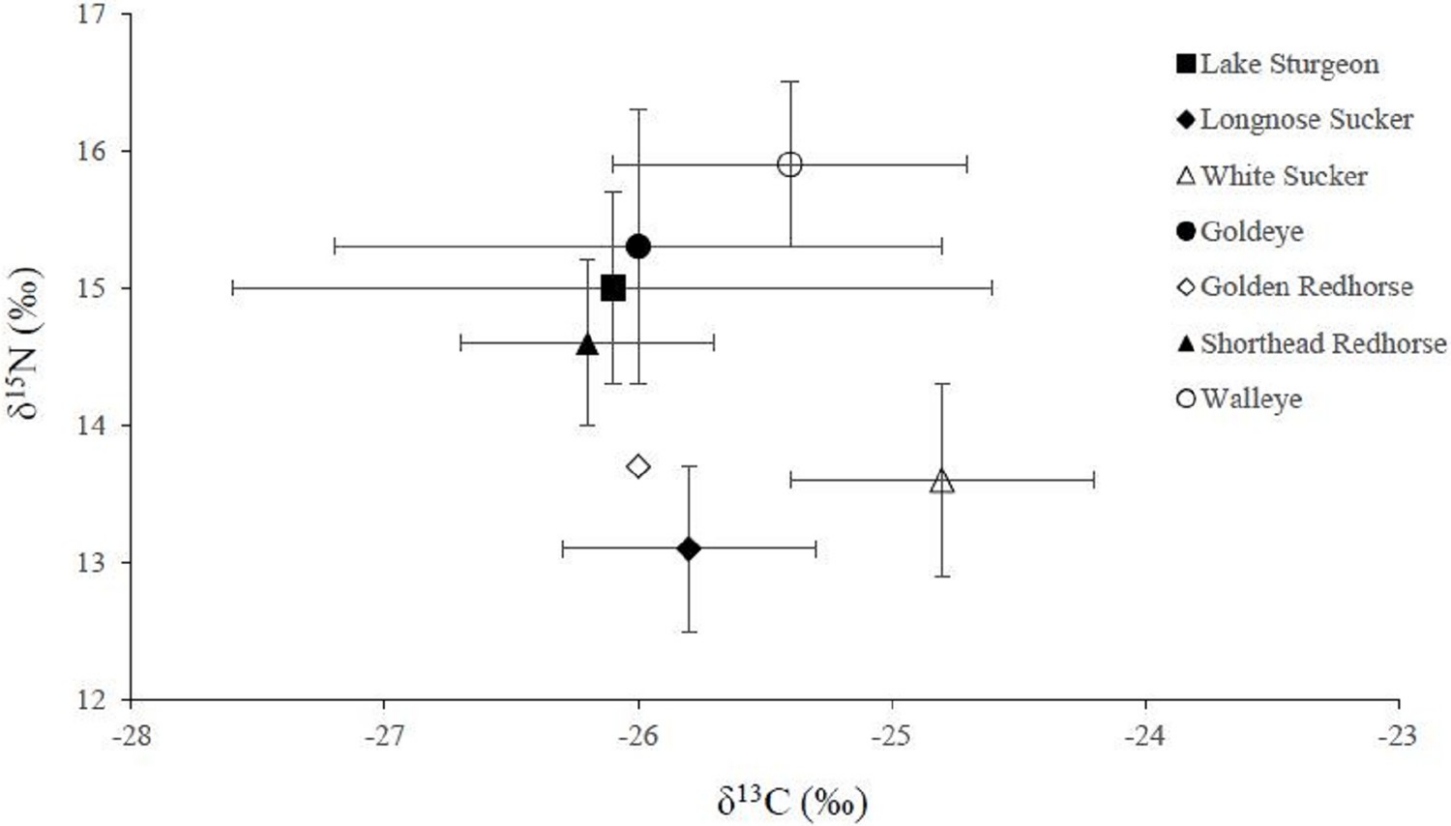
Mean δ^13^C (x-axis) and mean δ^15^N (y-axis) stable isotope values with standard deviations for lake sturgeon and companion fishes caught in the Saskatchewan River between May and October of 2011.

Five companion fish species were caught in 2011 (Table 3). Of the five, walleye had the highest mean δ^15^N value (15.9 ± 0.55‰); and longnose sucker and white sucker had the lowest (13.1 ± 0.55‰ and 13.6 ± 0.71‰, respectively) (Table 3). Despite the overlap in δ^15^N values between longnose sucker and white sucker, there was minimal overlap in their δ^13^C values (-25.8 ± 0.50‰ and -24.8 ± 0.56‰, respectively). Further, white sucker had the highest δ^13^C value and shorthead redhorse the lowest (-26.2 ± 0.54‰). Goldeye’s δ^13^C values were most variable of the companion fishes (-26.0 ± 1.15‰). During the same time period, we caught a total of 104 lake sturgeon and there were no significant differences between juvenile and adult lake sturgeon δ^13^C and δ^15^N. The overall mean δ^13^C was -26.1 ± 1.5‰ and mean δ^15^N was 15.0 ± 0.7‰ (Fig 4).

**Table 3:** Sample size and mean δ^13^C and δ^15^N values with standard deviation, for all lake sturgeon and companion fishes collected in 2011. Lake sturgeon values represent a combination of adult and juveniles since they were not significantly different, and combined for diet analysis in the mixing model. Companion fishes were identified to species.

Fish species	Sample size	Mean corrected δ^13^C value with standard deviation (‰)	Mean δ^15^N value with standard deviation (‰)
Lake sturgeon	104	-26.1 ± 1.5	15.0 ± 0.7
Longnose sucker	11	-25.8 ± 0.50	13.1 ± 0.55
White sucker	14	-24.8 ± 0.56	13.6 ± 0.71
Goldeye	12	-26.0 ± 1.15	15.3 ± 1.01
Golden redhorse	1	-26.0	13.7
Shorthead redhorse	3	-26.2 ± 0.54	14.6 ± 0.56
Walleye	20	-25.4 ± 0.68	15.9 ± 0.55

## Discussion

In this study, we determined that both juvenile and adult lake sturgeon prey in the Saskatchewan River predominantly consisted of a fairly specific diet of crayfish and snails, while other benthic invertebrate taxa had only minimal contributions. Further, our analyses of companion fishes showed that lake sturgeon were the top predator of the benthic food web without any congener competing for the same primary food sources. Additionally, walleye which was more reliant on pelagic prey, appeared to be the overall top predator in this system. Finally, lake sturgeon carbon and nitrogen stable isotope values varied significantly among years and months, which was largely reflective of temporal hydrological patterns.

### Temporal and spatial shifts in lake sturgeon stable isotope values

Demonstrated in [23], a depletion of δ^13^C in aquatic primary producers occurs at higher flow velocities due to a reduced boundary layer around the substrate that limits CO_2_ diffusion / availability. Thus, during years of drought, low rainfall, or minimal runoff, less water enters the watershed and subsequent lower current velocities can increase δ^13^C values throughout the entire food web because of the change at the primary production level. Conversely, years with above average precipitation and runoff can decrease δ^13^C values throughout the system. Therefore, climate variability in correlation with current velocities can cause annual fluctuations in stable isotopes values of riverine flora and fauna. A previous study in Lake Diefenbaker, a large riverine reservoir along the South Saskatchewan River, identified the influence of seasonal and inter-annual discharge on δ^13^C values of Dissolved Inorganic Carbon (DIC) from 2010 to 2013 [39]. For most years, δ^13^C_DIC_ values decreased from May to August, as flow velocity declined after snow melt. Also, 2011 was an extreme discharge year, followed by 2013, 2012 and 2010, which had average discharge. Over the sampling period, average discharge by year was highly negatively correlated with δ^13^C_DIC_ values (*r*^2^ = 0.87, n = 4). Similarly, average summer discharge in our sampling region (confluence of North and South Saskatchewan River) was strongly negatively correlated with δ^13^C values of sturgeon tissue for the respective years (*r*^2^ = 0.99, n = 3). Hence, the observed variability of δ^13^C in lake sturgeon was most likely related to discharge patterns of the Saskatchewan River.

An alternative factor influencing seasonal δ^13^C changes in lake sturgeon could be opportunistic feeding on food items that are only temporarily available. For example, in the St. Lawrence Estuarine Transition Zone, lake sturgeon fed heavily on fish eggs in June [17]. This may be occurring in our study area since several spring spawning fish species inhabit the Saskatchewan River [4, 40]. Yet, consumption of eggs is not a universal food source across all lake sturgeon. Found by [13], only 12 of 200 lake sturgeon stomachs contained eggs. Regardless, it remains unclear if fish eggs contributed to lake sturgeon diets in our study since benthic sampling was limited to August 2010 when no fish species were spawning [4, 40]. Nevertheless, the strong association between river discharge and δ^13^C of lake sturgeon makes it unlikely that fish eggs were responsible for the seasonal variation in δ^13^C.

### Dietary proportion

Crayfish had the greatest contribution to juvenile and adult lake sturgeon diets in our study. This could be in part due to virile crayfish’s preference towards rocky bottom habitats [41] and the fact that all of our study sites in the Saskatchewan River contained this type of habitat. Moreover, competition between crayfish for preferred habitat may result in dislodgment from refuge sites, making crayfish more vulnerable to predation [42]. Further, crayfish are usually the largest aquatic invertebrate in temperate freshwater ecosystems, offering a greater foraging reward than smaller invertebrates [43, 44]. Gape size can be a limitation when consuming larger prey such as crayfish. But based on their large size and similarity in diet, neither juveniles nor adult sturgeon appeared to be limited by gape size. Similar to our study, crayfish were also identified as a main food source in the Kenogami River, Ontario [45], Lake Winnipeg [46], Lake of the Woods [16], and other locations across Ontario [13]. While [45] found increased predation pressure on crayfish with increasing sturgeon size (>800 mm), we did not detect any ontogenetic shifts between juvenile and adult lake sturgeon.

Similar to crayfish, snails inhabit a wide range of habitats spanning both aphotic and photic zones, feeding on detritus and periphyton covering boulders, cobble, or macrophytes [47, 37]. Hence, substantial snail contributions to sturgeon diets may be attributed to habitat overlap and feeding strategies that make them vulnerable to predation. In addition, snails may be more prevalent in sturgeon diets than other molluscs, due to their shells being smaller and more fragile compared to the mussel species collected. Smaller and more fragile shells may be easier for lake sturgeon to consume, crush, and digest; leading to selective feeding behaviour towards snails and away from mussels [48].

Previous studies have identified smaller mollusc taxa such as *Sphaerium* (*Sphaerium sp*. [Scopoli, 1777]), *Pisidium* (*Pisidium sp*. [Pfeiffer, 1821]), and zebra mussels (*Dreissena polymorpha* [Pallas, 1771]) as valuable food sources for lake sturgeon, but not the larger species identified in our study [14, 17, 36, 49]. Presumably, larger molluscs exceed lake sturgeon’s mouth gape diameters, making them unable to encapsulate their shells. Lake sturgeon mouth gape is measured as 70% of the inter-orbital width; the distance between their eyes [50]. From observations in the field (Braun, pers. observation), interorbital lengths rarely exceeded 100 mm, meaning, that maximum gape size was limited to 70 mm. Hence, lake sturgeon may be able to consume smaller mussels, such as *L*. *siliquoidea* (62.9 ± 20.2 mm) or juvenile *P*. *grandis* and *L*. *complanata*, but not their adults. However, juvenile mussels are intuitively more difficult for benthic surface feeding fish, such as lake sturgeon, to locate and consume since they bury themselves deeper into the sediment than adults [51].

Unlike previous studies [11, 12, 14, 17, 18, 19, 38, 52, 53], chironomids were almost completely absent from lake sturgeon diets in our study. This could be due to lake sturgeon not accessing profundal habitats [6] where the majority of the chironomid samples were collected. Despite varying feeding strategies displayed among chironomid species dependent on habitat and sediment composition [54], no significant difference in δ^13^C values were found between chironomids collected from Codette reservoir sites and riverine sites. Thus, the chironomid stable isotope values included in our diet analysis accurately represented chironomids at the sturgeon sampling sites, supporting our conclusion that lake sturgeon are not heavily foraging on them even when their habitats overlap.

Similar to chironomids, otherwise common lake sturgeon food sources such as caddisflies and mayflies also showed no significant differences between sampling locations and were only of minor importance in our study [7, 11, 12, 14, 15, 17, 55]. The relative absence of these genera from lake sturgeon diet may be associated with the preference of caddisflies and mayflies for shallow (< 2 m) habitats with fine substrate situated along the banks of lotic sites [33, 37]. This type of habitat is generally not utilized by lake sturgeon that are typically restricted to depths between 2 and 10 m year-round [56]. Therefore, the concentration of caddisflies and mayflies in shallower portions of the ecosystem minimized overlap with primary lake sturgeon habitat, decreasing their relevance as a food source. Furthermore, with no significant differences between mayflies from lentic and lotic habitats, or between habitats with varying substrate, suggests even mayfly families found in the Saskatchewan River systems associated with rocky habitats, such as Baetidae and Heptageniidae, could only minimally contribute to lake sturgeon diets [47, 57]. Since lake sturgeon are able to feed continuously, they may consume larger quantities of caddisflies and mayflies when migrating through shallow sections of the river [50]. In fact, caddisflies and mayflies were observed in lake sturgeon stomachs in a section of the Saskatchewan River in Alberta, Canada, primarily in shallower habitats with finer substrates than in our study [14]. However, absence or limited contribution of caddisflies and mayflies to lake sturgeon diet is not uncommon, as similar results were observed in Oneida Lake, New York and the St. Lawrence River Estuarine Transition Zone [36, 49].

### Ontogenic dietary shifts

Del^15^N differences between our age groups were negligible and percentages of prey reliance overlapped, thus, we conclude that crayfish and snails are critical food sources for both juvenile and adult lake sturgeon. Previous studies have also identified similar prey preferences across lake sturgeon life stages [52, 11, 12]. Additionally, [58] showed sturgeon as small as 75 mm preyed on crayfish. Yet, selective predation based on age class has been noted in other lake sturgeon populations. In the St. Lawrence Estuarine Transition Zone lake sturgeon over 600 mm routinely preyed on molluscs, while they were nearly absent in the diets of smaller lake sturgeon (<600 mm) [36]. Zebra mussels were the most frequent food source for lake sturgeon >900 mm in Oneida Lake, New York [49] (which to this point have not invaded the Saskatchewan River system). A shift from insect larvae to bivalves was observed in the St. Lawrence River at 700 mm [53], and a preference for crayfish in lake sturgeon >800 mm was documented in the Kenogami River, Ontario, while they primarily fed on trichoptera and odonata at shorter lengths [45]. Also [17] identified, that as lake sturgeon size increased there was a positive trend in mollusc consumption, while dipterans were consumed by lake sturgeon between 250–649 mm. Although ontogenetic shifts have been noted elsewhere, our results suggest consumption patterns in the Saskatchewan River are independent of age and size.

### Substrate influence on prey

The dominance of cobble and boulder substrate in lake sturgeon habitats most likely had an influence on lake sturgeon diets. In fact, the prey items with the largest diet contributions in our study were commonly associated with rocky habitats. Crayfish utilize rock crevices for protection and an ambush site when feeding, and snails rely on periphyton growing on boulders and cobble [37, 41, 42]. Furthermore, lake sturgeon inhabiting boulder- and cobble-dominated habitats show a greater propensity to feed on mussels and crayfish over caddisflies, chironomids, and mayflies [13]. However, [19] showed diets of juvenile sturgeon with access to both rocky and fine substrata habitats was dominated by chironomid species associated with fine sediment, while chironomid and mayfly species associated with the rocky habitats were not important. Moreover, several studies have shown when lake sturgeon feed primarily in locations with smaller particles, such as clay, sand, or silt; diets tend to be dominated by caddisflies, chironomids, and mayflies [11, 12, 13, 14, 18,19]. This is not surprising because fine sediment habitats tend to have higher abundances of chironomids, mayflies, and caddisflies (particularly chironomids in soft sediment profundal zones) [54, 59, 60]. Further, tube-dwelling chironomids, like *Chironomus sp*., express feeding behaviours that draw them out of their tube, potentially making them more vulnerable to predation by predators that cannot invade their tubes [19, 61]. Thus, the type of foraging behaviour of prey appears to have a critical impact on the prey type preferred by a specific lake sturgeon population, and our findings further corroborate that lake sturgeon consumption patterns are highly dependent on habitat and type of benthic fauna occupying the substrate.

### Food web configuration

Lake sturgeon had the highest δ^15^N values among benthivorous fishes (longnose sucker, shorthead redhorse, and white sucker), suggesting it occupies the position of top predator within the benthic food web. The two sucker species had the lowest δ^15^N values, while shorthead redhorse was intermediate. These three species have been shown to feed heavily on benthic invertebrates (eg. Chironomidae, Epheromoptera, molluscs, and Trichoptera) [56] and the comparatively low δ^15^N reflects the absence of higher trophic level diet (i.e., crayfish). Accordingly, benthivorous fishes other than lake sturgeon were consuming invertebrates that make up only a small portion of lake sturgeon diets. Thus, there appears to be little to no competition between shorthead redhorse, longnose sucker, and white sucker for dietary sources with lake sturgeon. Yet, based on their stable isotopic values, goldeye appear to have a similar diet to lake sturgeon. Goldeye typically feed opportunistically on the most abundant prey at the surface or sub-surface during the summer months [56]. Important food sources for goldeye are corixids (Hemiptera: Corixidae), Coleoptera, zooplankton (Cladocera [Latreille, 1829]), chironomids, larval fish, and a wide array of aquatic and terrestrial insects [62, 63, 64, 65]. Therefore, potential dietary overlap between lake sturgeon and goldeye may be attributed to the absence of otherwise common prey sources in our study sites. There has been no previous documentation of goldeye consuming crayfish, which would be highly unlikely due to their feeding strategy. Therefore, the δ^13^C values for goldeye in this study most likely reflect food sources that were not included such as zooplankton, Coleoptera, and corixids, rather than crayfish.

Among the fishes collected, walleye was the top predator, consuming prey with δ^15^N values higher than those of benthic invertebrates collected in this study. Typically, walleye are highly piscivorous and therefore their higher δ^15^N values likely reflect piscivory [56]. A largely fish-based diet for walleye suggests no direct competition with lake sturgeon for primary prey items. However, other piscivorous species in the Saskatchewan River system (burbot (*Lota lota* [Linnaeus, 1758]), northern pike (*Esox lucius* [Linnaeus, 1758]), and sauger (*Sander canadensis* [Griffith and Smith, 1834]) that were not included in this study, may directly compete with either, or both, walleye and lake sturgeon for preferred prey [11, 40, 56]. In fact, [11] found burbot diets to significantly overlap with lake sturgeon in a system where lake sturgeon fed primarily on chironomids and mayflies. In addition, they also found burbot consumed crayfish and fish, while walleye and northern pike fed heavily on smaller fish species and crayfish. Yet, in the Winnipeg River Reservoirs and Round Lake, Manitoba none of the companion fishes fed on crayfish when co-occurring with lake sturgeon [66]. Thus, a similar scenario may be occurring in the Saskatchewan River where lake sturgeon are occupying a trophic niche that is not used by other predatory fish. However, it would be fallacious to assume that there is no competition for critical food sources between lake sturgeon and other predatory fish without a more detailed analysis. Furthermore, a more in-depth interpretation of lake sturgeon niche position should be done by increasing congener sample sizes, sampling all predatory fish species with overlapping distributions, collecting more potential invertebrate prey taxa representing different trophic guilds, and subsequently applying ecological niche and niche overlap determining Bayesian statistical models [21, 67, 68, 69].

### Conservation implications

Our study, as well as [6], shows that within the Saskatchewan River system, lake sturgeon rely heavily on lotic rocky pools, and not the deeper lentic habitats formed by the Francis-Finlay Dam. This is not surprising because dam-influenced alterations to river habitat often represent a disadvantage to lotic species, making unaltered, upstream lotic habitat highly valuable to lake sturgeon. Generally, raised water levels and restricted water flow from inundation leads to increased sedimentation and loss of turbulent reaches crucial for a viable lake sturgeon population [70]. A reduction in turbulent reaches removes potential spawning locations; and, infills rock crevices by increasing sedimentation, eliminating refuge sites (crayfish) and anchoring points (snails) for important prey, and protection from predation on lake sturgeon eggs [56]. Additionally, dams may restrict fish migration, and the ambiguity of spawning locations throughout our section of the Saskatchewan River would potentially prevent access for lake sturgeon to vital habitat if more dams were constructed. Thus, lake sturgeon survival in the Saskatchewan River is tied to the prolonged existence of these types of lotic habitats and accessibility to them. Elimination of lotic habitats would also impact lower trophic levels, but the impact on lake sturgeon would probably be more severe as higher trophic levels are disproportionately more susceptible to habitat disturbances [71]. Therefore, a conservation strategy is needed that will limit/prevent further degradation to the Saskatchewan River. Of the conservation strategies available, potentially the most applicable and feasible would be to avoid further habitat fragmentation via new dams and preserve existing habitat. Habitat preservation is a proven conservation strategy that not only protects the habitat for species of interest, but also critical prey items [72]. If habitat preservation alone doesn’t prove effective, habitat improvement methods, such as artificial spawning reefs, could be investigated and considered as a plausible conservation strategy to increase spawning or other important habitats for lake sturgeon [73]. For the Saskatchewan River system, persevering rocky lotic pools would significantly increase the likeliness of a sustained lake sturgeon population.

## Supporting information

S1 Table (XLSX)
